# Comparative Analysis of the Cytotoxic Effects of Okadaic Acid-Group Toxins on Human Intestinal Cell Lines

**DOI:** 10.3390/md12084616

**Published:** 2014-08-21

**Authors:** Pierre-Jean Ferron, Kevin Hogeveen, Valérie Fessard, Ludovic Le Hégarat

**Affiliations:** ANSES, Fougères Laboratory, Toxicology of Contaminants Unit, French Agency for Food, Environmental and Occupational Health & Safety, Fougères 35302, France; E-Mails: pierre-jean.ferron@anses.fr (F.P.-J.); kevin.hogeveen@anses.fr (H.K.); valérie.fessard@anses.fr (F.V.)

**Keywords:** high content analysis, okadaic acid, dinophysistoxins, apoptosis, γ-H2AX, inflammation, proliferation

## Abstract

The phycotoxin, okadaic acid (OA) and dinophysistoxin 1 and 2 (DTX-1 and -2) are protein phosphatase PP2A and PP1 inhibitors involved in diarrhetic shellfish poisoning (DSP). Data on the toxicity of the OA-group toxins show some differences with respect to the* in vivo* acute toxicity between the toxin members. In order to investigate whether OA and congeners DTX-1 and -2 may induce different mechanisms of action during acute toxicity on the human intestine, we compared their toxicological effects in two* in vitro* intestinal cell models: the colorectal adenocarcinoma cell line, Caco-2, and the intestinal muco-secreting cell line, HT29-MTX. Using a high content analysis approach, we evaluated various cytotoxicity parameters, including apoptosis (caspase-3 activation), DNA damage (phosphorylation of histone H2AX), inflammation (translocation of NF-κB) and cell proliferation (Ki-67 production). Investigation of the kinetics of the cellular responses demonstrated that the three toxins induced a pro-inflammatory response followed by cell cycle disruption in both cell lines, leading to apoptosis. Our results demonstrate that the three toxins induce similar effects, as no major differences in the cytotoxic responses could be detected. However DTX-1 induced cytotoxic effects at five-fold lower concentrations than for OA and DTX-2.

## 1. Introduction

Okadaic acid (OA) is a toxin involved in diarrhetic shellfish poisoning (DSP) syndrome, causing gastro-intestinal symptoms following ingestion of contaminated shellfish [1,2]. OA and its analogues, the dinophysistoxins (DTXs), are polyether toxins synthesized by dinoflagellates, such as *Dinophysis* and *Prorocentrum* [3]. OA is the major DSP toxin found in Europe, while the methylated derivative, dinophysistoxin-1 (DTX-1), has been mainly found in Japan [4]. Some European countries (Ireland, Italy, Spain and Portugal) have also detected DTX-1 and the isomeric analogue DTX-2 in shellfish [5,6]. DTX-3 is the 7-*O*-acyl fatty acid ester analogue of OA, DTX-1 and DTX-2 formed by shellfish metabolism [7]. After consumption, DTX-3 is hydrolyzed in the human stomach, releasing the parent toxin [8].

The acute* in vivo* toxicity of these toxins has been primarily investigated by intraperitoneal injection in mice, and similar lethal dose 50 (LD_50_) values are observed for DTX-1 and OA, while DTX-2 is about 0.6-times as potent [9]. The European Food Safety Authority (EFSA) has determined toxicity equivalent factors (TEFs) of one for the reference toxin, OA, and DTX-1 and 0.6 for DTX-2 [10]. 

OA and its analogues inhibit serine/threonine protein phosphatases 1 (PP1) and 2A (PP2A) [11,12], which are essential in the regulation of intracellular processes in mammalian cells [13]. However, the structural differences between OA and its analogues with respect to the number and position of methyl groups have been shown to modulate the affinity of the toxin for the catalytic site of PP2A [14,15]. Nevertheless, it has been suggested that the difference in PP2A affinity cannot fully explain the difference in toxicity between the congeners of the OA family [16]. 

Several key mechanisms of toxicity have already been reported for OA, including disruption of the cell cycle [17], induction of cell proliferation [18,19], DNA damage [20,21], apoptosis [11], as well as an inflammatory response [22]. However, only limited studies have seriously investigated the toxic responses of the DTX-1 and DTX-2 analogues. Recently, Rubiolo* et al*. have shown that OA and DTX-2 induce similar effects in human primary hepatocytes, albeit with different potencies [23]. Fernandez* et al*. have demonstrated a lack of cytotoxicity of OA and analogues in differentiated Caco-2 cells [24], whereas proliferating Caco-2 cells are considerably more sensitive to the effects of OA, as shown by Del Campo and Serandour [25,26]. However, available data comparing the toxicity of the three congeners in* in vitro* studies is lacking. 

In order to compare the toxic effects of the OA analogues, we chose to investigate the acute* in vitro* toxicity of OA, DTX-1 and DTX-2 in the human intestinal cell lines, Caco-2 and HT29-MTX, since the intestine is the major target of OA and its analogues. Caco-2 cells are widely used as an enterocyte model system in* in vitro* toxicology [27,28], whereas HT29-MTX cells have muco-secretant characteristics [27]. Both cell lines were used in a proliferative state, mimicking intestinal epithelial cell turnover and to study the effect of the toxins on cell proliferation. 

Multiparametric fluorescence microscopy was chosen in the present study to measure cytotoxic parameters in individual cells through image analysis using a high content analysis (HCA) approach [29,30]. This novel technology has been successfully applied in the assessment of toxicity of numerous compounds, including pharmaceuticals and environmental contaminants. As several endpoints are measured at the same time, it is a powerful tool to investigate the pathways involved in toxicity. Our study is the first dedicated to the investigation of the toxicity of marine toxins using this innovative approach. In the current study, four suitable endpoints were selected: Ki-67 as a marker of cell cycle disruption [31], activation of caspase-3 as an apoptotic marker [32], phosphorylation of histone H2AX as marker of DNA double-strand breaks [33] and, finally, the translocation of NF-κB as a marker of the cellular inflammatory response [34].

## 2. Results and Discussion

### 2.1. Neutral Red Uptake Assay

**Figure 1:**
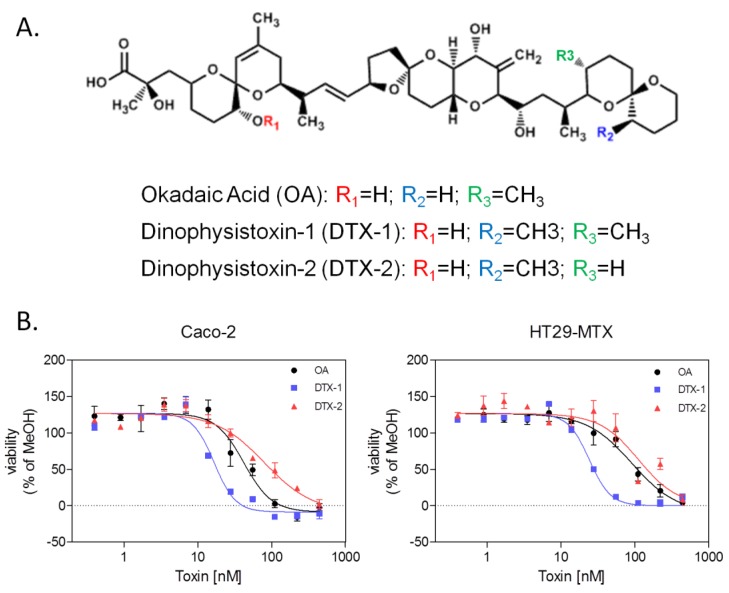
Structure of okadaic acid (OA), dinophysistoxin-1 (DTX-1) and -2 and their cytotoxicity in Caco-2 and HT29-MTX cells measured by the neutral red uptake assay. (**A**) Chemical structure of OA, DTX-1 and DTX-2; (**B**) Dose response curves represent the viability of Caco-2 and HT29-MTX cells compared to vehicle control conditions (5% MeOH) (24-h treatment).

The cytotoxicity of OA, DTX-1 and DTX-2 was evaluated in proliferating Caco-2 and HT29-MTX cells with the neutral red uptake (NRU) assay. After 24 h of treatment, a decrease in cell viability was observed for all toxins (Figure 1B), with DTX-1 being significantly more toxic in the two cell lines (IC50 of 22 nM). Treatment with OA resulted in IC_50_ values of 49 nM in Caco-2 cells and 75 nM in HT29-MTX cells. DTX-2 was the least toxic toxin with an IC_50_ of 106 nM in Caco-2 cells and 213 nM in HT29-MTX cells (Table 1). 

**Table 1:** IC_50_ values of OA group toxins in Caco-2 and HT29-MTX cells calculated from the neutral red uptake assay. IC_50_ values and 95% CI were calculated from three independent experiments performed in triplicate.

Toxins	Caco-2		HT29-MTX	
	IC_50_ [nM]	95% CI	IC_50_ [nM]	95% CI
OA	49.67	16.59–82.75	75.33	48.54–102.1
DTX-1	22.50	8.83–36.16	22.00	10.62–33.38
DTX-2	106.00	21.54–190.5	213.3	49.28–377.4

Rubiolo* et al*. have previously demonstrated that DTX-2 is less cytotoxic than OA in rat hepatocytes, although the difference is relatively minute [23]. Recent studies have demonstrated a lack of cytotoxic effects in differentiated Caco-2 cells treated with up to 1 mM OA, DTX-1 and -2, suggesting that proliferative cells, such as intestinal stem cells, are more sensitive to phosphatase inhibitor compounds when compared to quiescent cells, such as differentiated enterocytes. Moreover, this difference in sensitivity between the proliferative and differentiated Caco-2 models demonstrates the importance of assessing cytotoxicity in both models in order to characterize specific cell-type toxicity, since the intestinal epithelium contains both proliferative and non-proliferative cells. Based on these results, three doses for each toxin were selected for HCA studies with undifferentiated Caco-2 and HT29-MTX cells: 50, 100 and 150 nM for OA and DTX-2 and 10, 20 and 30 nM for DTX-1. 

### 2.2. Cell Cycle Disruption

**Figure 2:**
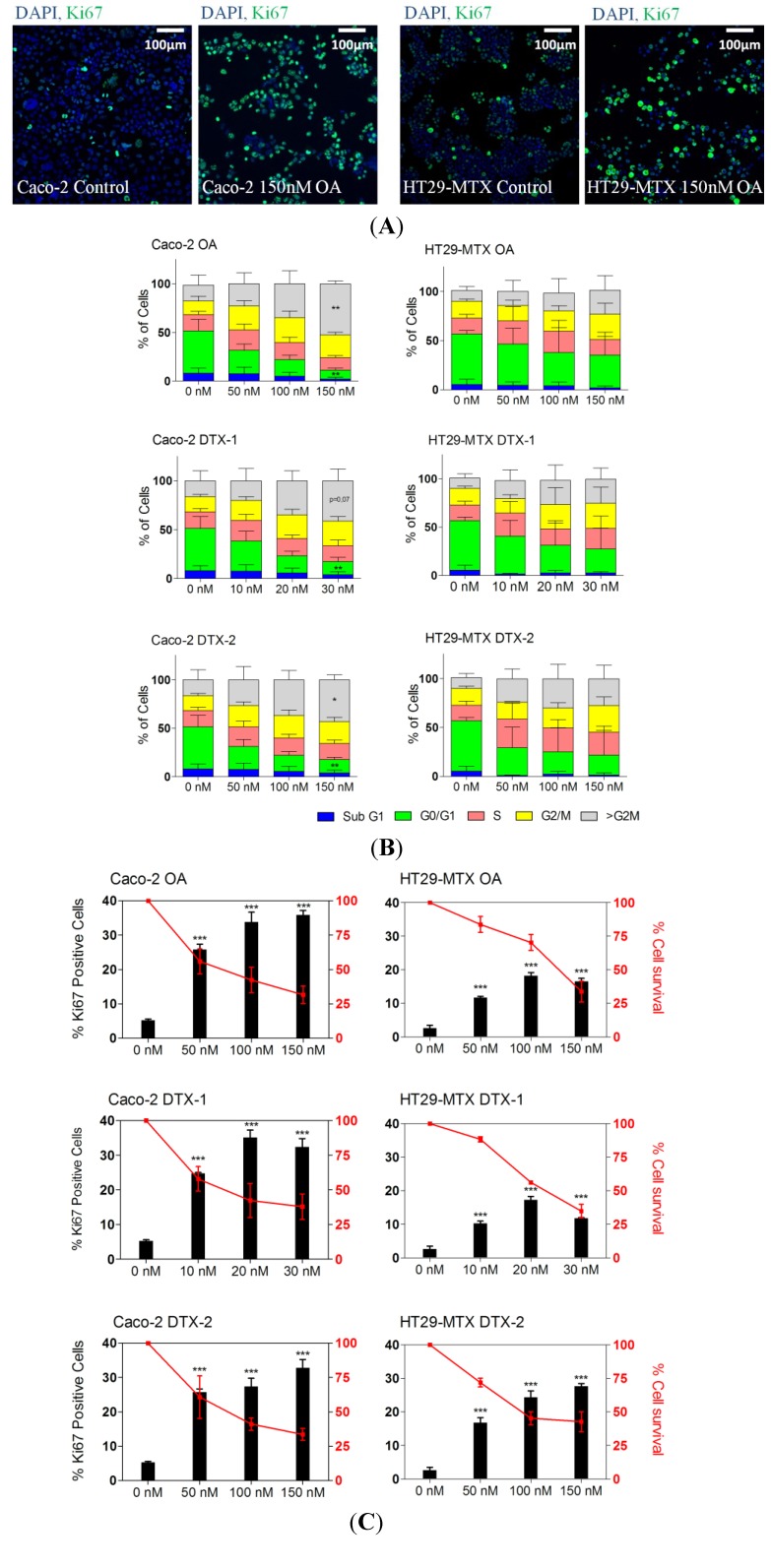
The effect of OA, DTX-1 and -2 on the cell cycle and the percentage of Ki-67-positive cells in Caco-2 and HT29-MTX cells. Representative images at 10× magnification of Caco-2 and HT29-MTX cells treated 24 h with 150 nM OA (**A**); DAPI staining in blue corresponds to the nucleus, and Ki-67 is shown in green. Caco-2 and HT29-MTX cells were treated with OA, DTX-1 and -2 for 24 h (**B**). Cell viability was calculated compared to control conditions; the cell cycle was determined using nuclear DAPI fluorescence; (**C**) The percentage of Ki-67-positive cells was determined using a threshold from control data as indicated in the Materials and Methods section. All error bars denote the SEM of three independent experiments. Statistical significance is depicted as follows: *** *p* < 0.001.

Cells positive for the Ki-67 antigen are considered to be in an active phase of cell cycle [31]. This marker is commonly used to detect increases in cell proliferation and can be easily adapted to high content analysis. Figure 2 represents the cell cycle analysis and the percentage of Ki-67-positive cells in Caco-2 and HT29-MTX cells treated for 24 h with OA and DTX-1 and -2. Although a dose-dependent increase in Ki-67-positive cells was also observed in HT29-MTX cells, treatments induced fewer Ki-67-positive cells than in Caco-2 cells. The three toxins increased the percentage of Ki-67-positive cells in both intestinal cell models, indicative of a cell cycle disturbance. These results were confirmed by cell cycle analysis based on the quantification of DNA content through image analysis on the Arrayscan VTi. A dose-dependent modification of cell cycle phases in Caco-2 and HT29-MTX cells was observed. A dose-dependent decrease in G0/G1 and an increase in >G2M was observed for OA, DTX-1 and DTX-2. Nevertheless, Caco-2 cells were more affected by the DSP toxins than HT29-MTX cells. Although the induction of an aberrant cell cycle in mammalian cells by phosphatase inhibitors, including OA and DTX-2, has already been demonstrated [17,25,35], the present study demonstrates for the first time the effects of DTX-1 on the cell cycle. 

PP2A is involved in the regulation of the cell cycle and is particularly important for mitosis exit, leading to >G2M [14,18,36]. Interestingly, while the Caco-2 cell line is p53 deficient, HT29-MTX cells present an aberrant p53 function [37]. This difference in p53 status, thereby modulating cellular protection from the induction of an aberrant cell cycle [38], could explain the lower induction of >G2M and Ki-67-positive cells in HT29-MTX cells when compared to Caco-2 cells. Therefore, the considerable cell cycle modification observed in Caco-2 cells could be due to PP2A inhibition in the absence of the protective effect of p53. Overall, the potency of each toxin in terms of the Ki67 response in Caco-2 cells closely corresponds with their ability to inhibit PP2A (DTX-1 > OA > DTX-2) [14]. However, HT29-MTX cells appear to be more sensitive to DTX-2 than OA in terms of the Ki-67 response, suggesting a pathway of toxicity other than PP2A inhibition.

Recently, by comparing PP2A inhibition and* in vivo* and* in vitro* acute toxicities, Munday (2013) suggested that the inhibition of PP2A cannot fully explain the diarrhoeagenicity and intestinal effects observed with this group of toxins [16]. In addition to differences in the affinity for PP2A, differences in the intracellular bioavailability of the toxins could also explain the greater cytotoxicity of DTX-1. Indeed, it has been shown that an active transport efflux may be involved as an mechanism of excretion of OA and DTX-1 and -2 from intestinal cells [24,39]. Furthermore, OA, DTX-1 and DTX-2 have been shown to form metabolites, in particular due to CYP450 activity [40]. It has been reported that CYP450-OA metabolites have affinity for PP2A [41], and that bioactivation can enhance certain toxicological effects, including DNA damage. Therefore, low uptake in intestinal cell lines and differences in affinity of the three analogues for efflux transporters or metabolic enzymes could represent a possible explication for the differences in toxicity observed.

### 2.3. Inflammatory Response

**Figure 3:**
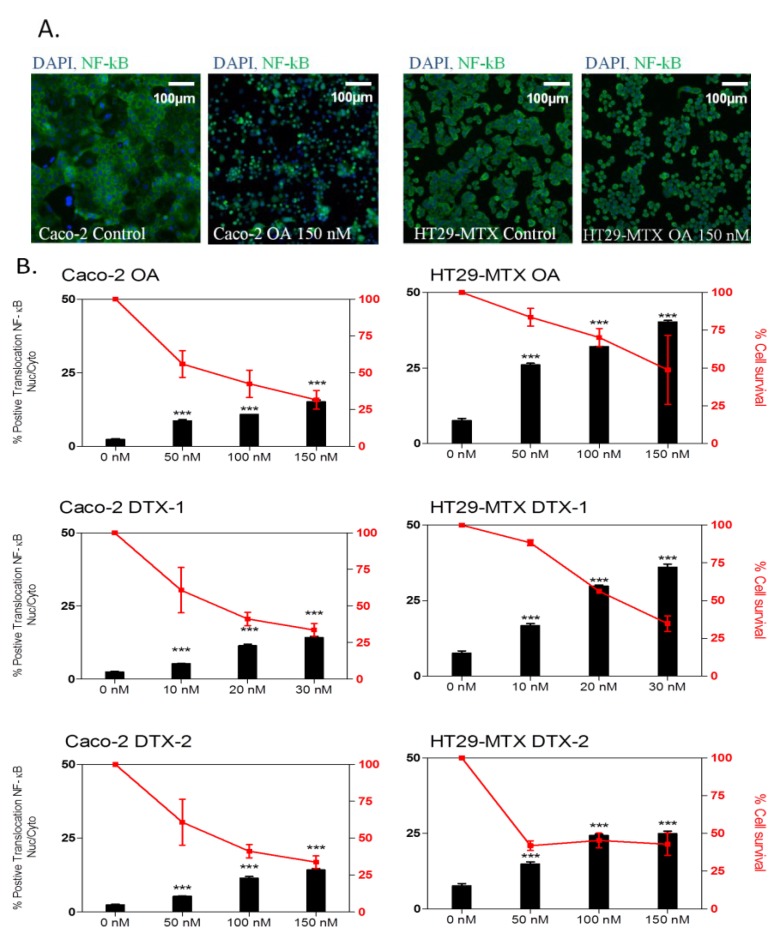
The effect of OA DTX-1 and -2 on NF-κB translocation in Caco-2 and HT29-MTX cells. Representative images at 10× magnification of Caco-2 and HT29-MTX cells treated 24 h with 150 nM OA (**A**); DAPI staining in blue corresponds to the nucleus, while NF-κB in green is present throughout the cell. Caco-2 and HT29-MTX cells were treated with OA, DTX-1 and -2 for 24 h and labeled for detection of NF-κB translocation by high content analysis (**B**). Cell viability was calculated compared to control conditions, and the percentage of positive cells for NF-κB translocation was determined with a threshold based on control conditions. All error bars denote the SEM of three independent experiments. Statistical significance is depicted as follows: *** *p* < 0.001.

The transcription factor, NF-κB, is involved in the pro-inflammatory immune response, as well as in the response to cellular stress [42]. NF-κB is located in the cytosol complexed with the inhibitory protein IκBα and, when activated through a PKAc-dependent mechanism, translocates to the nucleus, activating a variety of genes involved in the inflammatory response. The translocation of NF-κB from the cytoplasm to the nucleus was studied in Caco-2 and HT29-MTX cells treated for 24 h with OA, DTX-1 and -2. The NF-κB fluorescence (green) was mainly present in the cytoplasm of untreated cells, while a translocation from the cytoplasm to the nucleus was observed following treatment with OA (Figure 3A). The three toxins induced a dose-dependent response in both cell lines (Figure 3B). In Caco-2 cells, the three toxins induced similar increases in NF-κB translocation, while in HT29-MTX cells, DTX-2 induced a weaker response when compared to OA and DTX-1. In contrast to the Ki-67 response, a larger number of positive cells were observed in HT29-MTX cells when compared to Caco-2 cells for the same toxin concentration. In HT29-MTX cells treated with the highest concentration, DTX-2 induced 25% positive cells, whereas 40% NF-κB-positive cells were observed at the same concentration of OA. DTX-1 induced a similar response (up to 35%) when compared to OA, although at lower concentrations.

NF-κB activation can initiate both inflammatory and pro-survival signaling pathways in epithelial cell lines [43,44]. Significant intestinal inflammation is a key early response following an acute exposure to DSP toxins* in vivo* [45], and NF-κB has been shown to be involved in gut inflammation. Therefore, the rapid translocation of NF-κB at low toxin concentrations could provide a molecular explanation for the intestinal inflammatory response* in vivo*. Increases in interleukin-8 secretion in Caco-2 and HT29-MTX cells following treatment with OA, DTX-1 and DTX-2 (Ferron, unpublished data) provide supporting evidence for the involvement of inflammation in OA group toxicity.

### 2.4. DNA Damage

**Figure 4:**
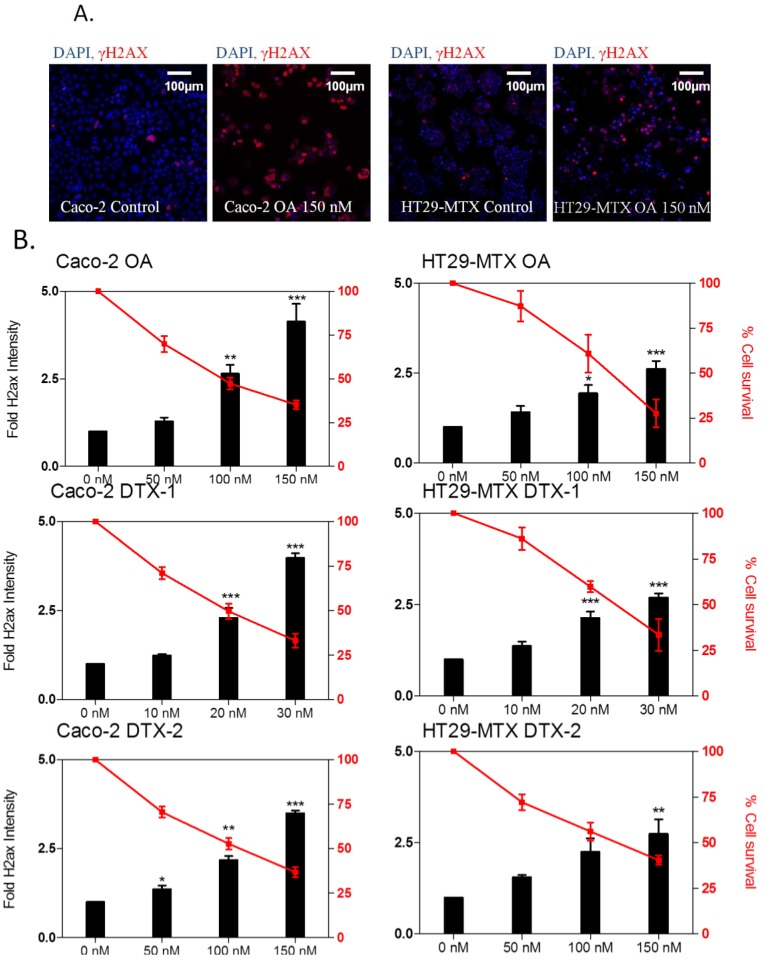
The effect of OA DTX-1 and -2 on the γH2AX response in Caco-2 and HT29-MTX cells. Representative images at 10× magnification of Caco-2 cells and HT29-MTX cells treated 24 h with 150 nM OA (**A**); DAPI staining in blue corresponds to the nucleus, while nuclear γH2AX is represented in red. Caco-2 and HT29-MTX cells were treated with OA, DTX-1 and -2 for 24 h and labeled for detection of γH2AX by high content analysis (**B**). Cell viability was calculated compared to control conditions, as was the fold of induction of γH2AX. All error bars denote the SEM of three independent experiments. Statistical significance is depicted as follows: * *p* < 0.05, ** *p* < 0.01, *** *p* < 0.001.

The phosphorylation of histone H2AX (γH2AX) is a marker of DNA double-strand breaks (DSBs). Following the induction of DNA damage (single breaks or DNA-adducts), DSBs formed due to the blockage of replication forks, resulting in H2AX phosphorylation, which permits a relaxing of the chromatin structure necessary to facilitate the recruitment of DNA repair enzymes. The fluorescence of γH2AX (in red) was detected in the nucleus of cells exposed to 150 nM OA (Figure 4A). Following a 24-h treatment, the three toxins induced dose-dependent increases in γH2AX fluorescence intensity in both cell lines (Figure 4B). Four-fold increases were observed for 150 nM OA and 30 nM DTX-1, and to a lesser extent (three-fold) with 150 nM DTX-2. Likewise, a similar dose response was observed in HT29-MTX cells, although the response of the three toxins was slightly lower than that of Caco-2 cells and did not exceed 2.5-fold compared to control cells. Although the ability of OA to induce damage to DNA is well known [20,21], a precise mechanism of action has not been identified. Previous studies have demonstrated that DNA damage induced by OA can occur through various mechanisms [46], including both aneugenic [47] and clastogenic effects [48]. However, since H2AX phosphorylation can also be a consequence of apoptosis [49], we have also examined whether the increase in γH2AX following treatment with toxins was correlated with apoptosis.

### 2.5. Apoptosis Induction

**Figure 5:**
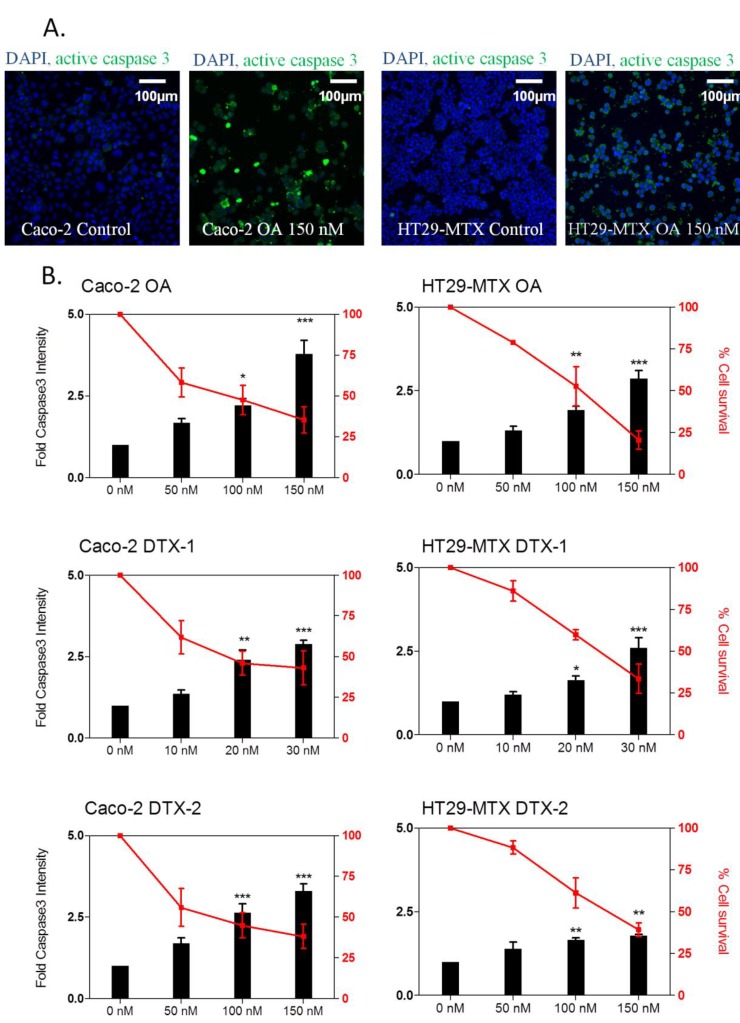
The effect of OA, DTX-1 and -2 on caspase-3 activation in Caco-2 and HT29-MTX cells. Representative images at 10× magnification of Caco-2 and HT29-MTX cells treated 24 h with 150 nM OA (**A**); DAPI staining in blue corresponds to the nucleus, while active caspase-3 in green is detected throughout the cell. Caco-2 and HT29-MTX cells were treated with OA, DTX-1 and -2 for 24 h and labeled for the detection of active caspase-3 by high content analysis (**B**). Cell viability was calculated compared to control conditions, as was the fold increase in active caspase-3 intensity. All error bars denote the SEM of three independent experiments. Statistical significance is depicted as follows: * *p* < 0.05, ** *p* < 0.01, *** *p* < 0.001.

Apoptosis is a complex process of programmed cell death mediated primarily through the activation of several caspase proteases [32]. Figure 5A illustrates the activation of caspase-3 in cells treated with 150 nM OA. Although no effect was observed at the lowest concentration, the three toxins induced apoptosis in a concentration-dependent manner in both cell lines (Figure 5B). In Caco-2 cells, OA induced a slightly greater effect than DTX-2 (four- and three-fold at 150 nM OA and DTX-2, respectively). DTX-1 induced a weaker increase (2.5-fold) than OA and DTX-2, although this effect was observed at a considerably lower concentration of toxin (30 nM). Interestingly, greater responses were observed in Caco-2 cells when compared to HT29-MTX cells. In HT29-MTX cells, OA induced the highest apoptotic response, followed by DTX-1 and DTX-2. The apoptotic potential of OA has been reported in many cell types [50,51]. OA has been reported to induce apoptosis through various pathways, including ROS/MAPK signaling [50], activation of double-stranded RNA-dependent protein kinase (PKR) [52] or activation of the Fas death receptor [53]. In this study, we show that, similar to OA, DTX-1 and DTX-2 also induce apoptosis in Caco-2 and HT29-MTX cells through a similar mechanism involving the activation of caspase 3.

**Figure 6:**
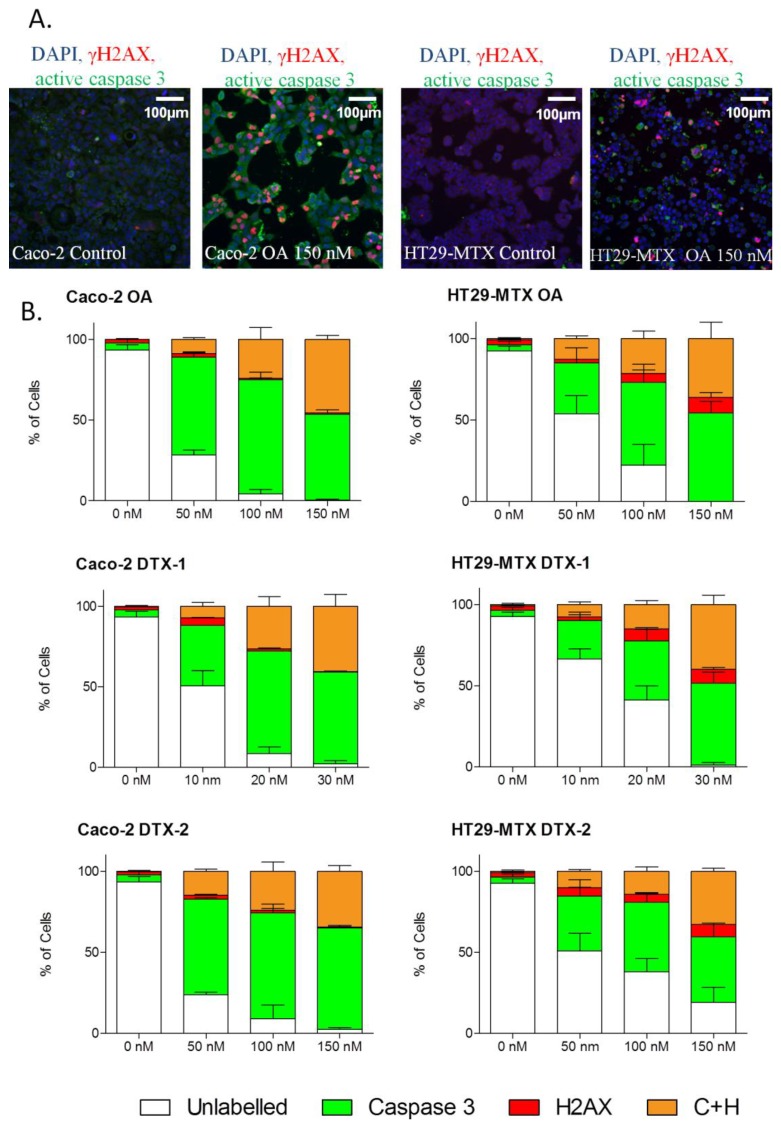
Induction of active caspase-3 and/or γH2AX by OA, DTX-1 and -2 in Caco-2 and HT29-MTX cells. Representative images at 10× magnification of Caco-2 and HT29-MTX cells treated 24 h with 150 nM OA (**A**); DAPI staining in blue corresponds to the nucleus. Caco-2 and HT29-MTX cells were treated with increasing concentrations of OA, DTX-1 and -2 for 24 h and were then labeled for detection of active caspase-3 (green) and γH2AX (red) by high content analysis (**B**). The percentage of positive cells for active caspase-3 and γH2AX was determined using a threshold based on control conditions. Data are expressed as stacked bars representing unlabeled cells (white), active caspase-3-positive cells (green), γH2AX-positive (red) and positive for both active caspase-3 and γH2AX (orange). *n =* 3.

### 2.6. γH2AX Related to Apoptosis

In order to discriminate whether the toxins induce DSBs as a result of clastogenicity or due to the induction of an apoptosis cascade [32,48,54], the percentage of γH2AX and caspase-3 co-labeled cells was quantified after a 24-h treatment with OA, DTX-1 and DTX-2 (Figure 6). We found that apoptosis appears to be the main cellular effect induced by the three toxins in Caco-2 and HT29-MTX cells. Secondly, although a large percentage of active caspase-3 cells was observed, we found that only a small proportion of cells were only γH2AX positive in response to OA and DTXs. Indeed, the majority of cells were co-labeled with the apoptotic caspase-3 marker, and the two markers appeared simultaneously after 24 h of treatment. Therefore, our results suggest that DSBs induced by DSP toxins are generated though an apoptotic response and not due to a genotoxic effect. 

**Figure 7:**
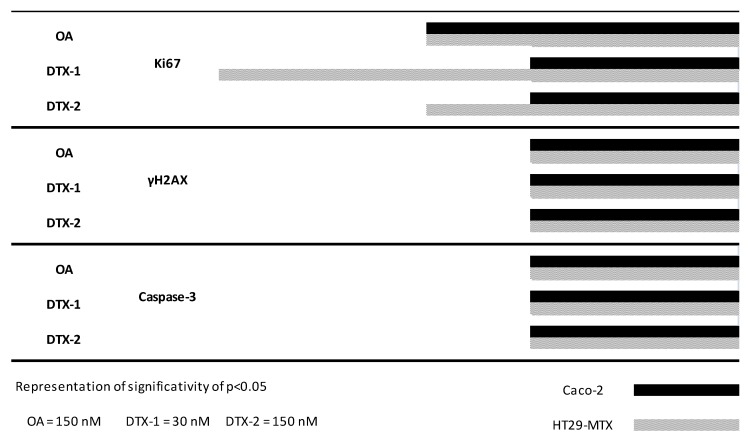
Kinetics of the detection of Ki-67, NF-κB, γH2AX and active caspase-3 in Caco-2 and HT29-MTX cells exposed to OA, DTX-1 and -2. Caco-2 and HT29-MTX cells were exposed to a single dose of OA (150 nM), DTX-1 (30 nM) and DTX-2 (150 nM) for different times. Each marker was analyzed in a separate experiment. Data are expressed as bars (solid for Caco-2 and striped for HT29-MTX) corresponding to a statistical significance of *p* < 0.05 (ANOVA analysis). Ki-67 production and NF-κB translocation were quantified as a percentage of positive cells compared to control conditions, while γH2AX and activation of caspase 3 were quantified as a fold change compared to control cells (*n =* 3).

### 2.7. Temporal Sequence in Cytotoxicity Responses

Time course studies were conducted in order to determine whether the toxins could be distinguished by the kinetics of induction of cellular events. In these experiments, cells were treated with the highest concentration of toxin for 4, 8, 16 or 24 h. After treatment, cells were labeled and quantified for the different markers. The times for which statistically significant positive responses were observed are summarized in Figure 7. 

With the exception of the Ki-67 response to DTX-1 and-2, all markers responded in a similar manner with respect to time in both Caco-2 and HT29-MTX cells. Although Ki-67 was induced very early (at 4 h) with DTX-1 in HT29-MTX cells, NF-κB translocation was the earliest detectable marker irrespective of the toxin, occurring as early as eight h following treatment with OA and 16 h following DTX-1 and -2 treatment. Finally, significant increases in γH2AX and active caspase-3 were observed only after a 24-h exposure to the three toxins. Results indicate that NF-κB translocation, known to be an early marker of the pro-inflammatory response, is the first effect detected after toxin treatment. Following this, the toxins induced a disturbance of the cell cycle leading to the activation of caspase-3 and the induction of DSBs.

The inhibition of PP2A (and to a lesser extent, PP1) is generally thought to represent the principal mechanism of OA toxicity. Indeed, PP2A is essential for a large number of protein kinase signaling pathways [55] and has been shown to be involved in the Ras/ERK pathway [56], as well as the p53/p21 response to DNA damage [57], both of which are involved in cancer promotion [58]. Moreover, inflammation, provoking chronic lesions and diseases, such as cancer, can also be a key effector in OA toxicity considering the involvement of the endogenous TNF alpha pathway in OA tumor promotion [11].

Based on our study, cytotoxic effects were observed at considerably lower concentrations for DTX-1 when compared to OA and DTX-2. Nevertheless, we have demonstrated that DTX-1 and DTX-2 induce the same markers of toxicity as their analogue OA, suggesting similar acute effects for the whole family. In order to determine if the mechanisms of toxicity of OA, DTX-1 and DTX-2 can be distinguished by unique pathways, a non-targeted transcriptomic approach could be performed. 

## 3. Experimental Section 

### 3.1. Cell Cultures

Caco-2 cells (American Type Culture Collection HTB-37, LGC Standards, Molsheim, France) were cultured in MEM medium containing 10% fetal calf serum (FCS), 1% non-essential amino acids, 50 U/mL penicillin and 50 μg/mL streptomycin. HT29-MTX cells (gift from Tecla Lesuffleur, INSERM U843, Paris, France) were cultured in DMEM medium containing 10% FCS, 50 U/mL penicillin and 50 μg/mL streptomycin. Media, serum, amino acids solution and antibiotics were all from Invitrogen (Saint Aubin, France).

For assays, Caco-2 and HT29-MTX cells were seeded at a density of 50,000 cells/cm^2^ and 90,000 cells/cm^2^, respectively, in Nunc 96-well thin bottom microplates (Thermo Scientific, Waltham, MA, USA) 24 h prior to incubation with the toxins. 

### 3.2. Toxins

Okadaic acid, DTX-1 and DTX-2 dissolved in methanol (MeOH) were purchased from IMB/NRCC (Halifax, Nova Scotia, Canada). For assays, toxins were prepared in culture medium without FCS. 

### 3.3. Neutral Red Uptake Assay

Neutral red powder (N4638) was purchased from Sigma Aldrich (Saint-Louis, MO, USA). Neutral red solution 0.1% was prepared in PBS and stored at 4 °C. Following treatment with toxins, cells were rinsed in PBS. Neutral red solution (100 μL) was added to each well and incubated 2 h at 37 °C. Cells were then rinsed in PBS, and 100 μL of solubilization solution (1% acetic acid in 50% ethanol) were added to each well. Absorbance was read at 540 nm, and viability was calculated as the percentage of mean absorbance (*n =* 3) to the vehicle control condition (5% MeOH). 

### 3.4. Immunofluorescence

Based on NRU results, a range of 3 doses was selected for each toxin in order to study the acute effects between inhibitory concentration (IC) 20 and IC_75_. The same range was used for OA and DTX-2 (50, 100 and 150 nM), whereas DTX-1 was tested at a lower range (10, 20 and 30 nM), due to its higher toxicity in both cell lines. For kinetics experiments, the effects were studied only with the highest dose after different time exposure (0, 4, 8, 16 and 24 h). After incubation with the toxins, cells were fixed with 4% paraformaldehyde in phosphate buffered saline (PBS) and permeabilized with 0.2% Triton X-100. Plates were then incubated in blocking solution (PBS with 1% BSA and 0.05% Tween-20) for 30 min before the addition of primary antibodies. All antibodies were prepared in blocking solution and filtered at 0.2 μm. The mouse monoclonal anti-γH2AX ser140 antibody (MA1-2022) was purchased from Thermo Scientific. The other primary and secondary antibodies were purchased from Abcam (Cambridge, UK): rabbit monoclonal anti active caspase-3 antibody (ab13847), rabbit monoclonal anti Ki-67 antibody (ab15580), rabbit monoclonal anti NF-κB antibody (ab16502), goat anti-Rabbit IgG DyLight^®^ 488 (ab96891) and goat anti-Mouse IgG H&L DyLight^®^ 550 (ab96876). Primary antibodies (1/1000) were incubated 1 h at room temperature. After washing with PBS + 0.05% Tween 20, secondary antibodies (1/1000) were incubated for 45 min at room temperature. Nuclear DAPI (1 μg/mL) staining was used for automated cell identification by high content analysis.

### 3.5. High Content Analysis

Plates were scanned with the Thermo Scientific ArrayScan VTI HCS Reader (Thermo Scientific, Waltham, MA, USA) and analyzed using the Target Activation module of the BioApplication software (Thermo Scientific, Waltham, MA, USA). For each well, 10 fields (10× magnification, white bar = 100 μm) were scanned and analyzed for quantification of immunofluorescence. Cell viability was determined by cell counting from DAPI staining and expressed as a percentage of cells compared to vehicle control condition. Nuclear DAPI staining was used to analyze cell cycle phases using the module Cell Cycle Analysis of the BioApplication software (Thermo Scientific, Waltham, MA, USA). Caspase-3 activation was quantified in the whole cell and expressed as a fold increase compared to untreated cells. γH2AX was quantified in cell nuclei and expressed as a fold increase compared to control cells. Nuclear Ki-67 intensity was quantified using a nuclear mask, while NF-κB translocation was calculated using a ratio of cytoplasm and nuclear fluorescence. Cells were defined as Ki-67-positive when fluorescence exceeded a threshold of 2 standard deviations when compared to the mean fluorescence observed in the control. Cells positive for NF-κB translocation were defined when the ratio of nuclear to cytoplasmic intensity exceeded a threshold of 2 standard deviations with respect to control conditions. Co-labeling experiments were also analyzed using a 2 standard deviations threshold to identify the different populations of responding cells.

### 3.6. Statistics

All experiments were repeated at least 3 times. Data were analyzed by one-way analysis of variance (ANOVA) followed by Dunnett’s *post hoc* tests using Prism 5 (GraphPad Software, Inc., La Jolla, CA, USA). All error bars denote SEM. Statistical significance was depicted as follows: * *p* < 0.05, ** *p* < 0.01, *** *p* < 0.001.

## 4. Conclusions

Our study provides* in vitro* results concerning the acute toxicity of DTX-1 and -2 in intestinal cells relative to OA. This study demonstrates that high content analysis on proliferating human intestinal cells provides a fast and reliable tool for studying and comparing the* in vitro* toxicity of OA and its analogues, DTX-1 and -2. Although no qualitative differences in the acute toxic events caused by OA and its analogues were observed, we found that DTX-1 was five-times more toxic than OA and DTX-2. Further investigation is therefore required to determine why DTX-1 induces toxicity at much lower concentrations when compared to OA and DTX-2. Several mechanisms, including the bioavailability and metabolism of the toxins, could play an important role and should be investigated in greater detail. Globally, the responses of Caco-2 and HT29-MTX cells to OA and analogues are similar, although different pathways may be involved.

## References

[1] Fujiki H., Suganuma M., Suguri H., Yoshizawa S., Takagi K., Uda N., Wakamatsu K., Yamada K., Murata M., Yasumoto T. (1988). Diarrhetic shellfish toxin, dinophysistoxin-1, is a potent tumor promoter on mouse skin. Jpn. J. Cancer Res. Gann..

[2] Murata M., Shimatani M., Sugitani H., Oshima Y., Yasumoto T. (1982). Isolation and structural elucidation of the causative toxin of the diarrhetic shellfish poisoning. Bull. Jpn. Soc. Sci. Fish..

[3] Vale P., Antónia M., Sampayo M. (1999). Esters of okadaic acid and dinophysistoxin-2 in Portuguese bivalves related to human poisonings. Toxicon.

[4] Suzuki T., Yasumoto T. (2000). Liquid chromatography–electrospray ionization mass spectrometry of the diarrhetic shellfish-poisoning toxins okadaic acid, dinophysistoxin-1 and pectenotoxin-6 in bivalves. J. Chromatogr. A.

[5] Carmody E.P., James K.J., Kelly S.S. (1996). Dinophysistoxin-2: The predominant diarrhoetic shellfish toxin in Ireland. Toxicon.

[6] Moroño A., Arévalo F., Fernández M., Maneiro J., Pazos Y., Salgado C., Blanco J. (2003). Accumulation and transformation of DSP toxins in mussels *Mytilus galloprovincialis* during a toxic episode caused by *Dinophysis acuminata*. Aquat. Toxicol..

[7] Terao K., Ito E., Yanagi T., Yasumoto T. (1986). Histopathological studies on experimental marine toxin poisoning. I. Ultrastructural changes in the small intestine and liver of suckling mice induced by dinophysistoxin-1 and pectenotoxin-1. Toxicon Off. J. Int. Soc. Toxinol..

[8] García C., Truan D., Lagos M., Santelices J.P., Díaz J.C., Lagos N. (2005). Metabolic transformation of dinophysistoxin-3 into dinophysistoxin-1 causes human intoxication by consumption of *O*-acyl-derivatives dinophysistoxins contaminated shellfish. J. Toxicol. Sci..

[9] Tubaro A., Giangaspero A., Ardizzone M., Soranzo M.R., Vita F., Yasumoto T., Maucher J.M., Ramsdell J.S., Sosa S. (2008). Ultrastructural damage to heart tissue from repeated oral exposure to yessotoxin resolves in 3 months. Toxicon.

[10] (2009). Marine biotoxins in shellfish—Summary on regulated marine biotoxins. EFSA J..

[11] Fujiki H., Suganuma M. (1993). Tumor promotion by inhibitors of protein phosphatases 1 and 2A: The okadaic acid class of compounds. Adv. Cancer Res..

[12] Takai A., Murata M., Torigoe K., Isobe M., Mieskes G., Yasumoto T. (1992). Inhibitory effect of okadaic acid derivatives on protein phosphatases. A study on structure-affinity relationship. Biochem. J..

[13] Cohen P.T. (1997). Novel protein serine/threonine phosphatases: Variety is the spice of life. Trends Biochem. Sci..

[14] Huhn J., Jeffrey P.D., Larsen K., Rundberget T., Rise F., Cox N.R., Arcus V., Shi Y., Miles C.O. (2009). A structural basis for the reduced toxicity of dinophysistoxin-2. Chem. Res. Toxicol..

[15] Larsen K., Petersen D., Wilkins A.L., Samdal I.A., Sandvik M., Rundberget T., Goldstone D., Arcus V., Hovgaard P., Rise F. (2007). Clarification of the C-35 stereochemistries of dinophysistoxin-1 and dinophysistoxin-2 and its consequences for binding to protein phosphatase. Chem. Res. Toxicol..

[16] Munday R. (2013). Is Protein phosphatase inhibition responsible for the toxic effects of okadaic acid in animals?. Toxins.

[17] Messner D.J., Ao P., Jagdale A.B., Boynton A.L. (2001). Abbreviated cell cycle progression induced by the serine/threonine protein phosphatase inhibitor okadaic acid at concentrations that promote neoplastic transformation. Carcinogenesis.

[18] Kurimchak A., Graña X. (2012). PP2A holoenzymes negatively and positively regulate cell cycle progression by dephosphorylating pocket proteins and multiple CDK substrates. Gene.

[19] Yuasa H., Yoshida K., Iwata H., Nakanishi H., Suganuma M., Tatematsu M. (1994). Increase of labeling indices in gastrointestinal mucosae of mice and rats by compounds of the okadaic acid type. J. Cancer Res. Clin. Oncol..

[20] Fessard V., Grosse Y., Pfohl-Leszkowicz A., Puiseux-Dao S. (1996). Okadaic acid treatment induces DNA adduct formation in BHK21 C13 fibroblasts and HESV keratinocytes. Mutat. Res..

[21] Le Hégarat L., Jacquin A.-G., Bazin E., Fessard V. (2006). Genotoxicity of the marine toxin okadaic acid, in human Caco-2 cells and in mice gut cells. Environ. Toxicol..

[22] Abolhassani M., Wertz X., Pooya M., Chaumet-Riffaud P., Guais A., Schwartz L. (2008). Hyperosmolarity causes inflammation through the methylation of protein phosphatase 2A. Inflamm. Res..

[23] Rubiolo J.A., López-Alonso H., Vega F.V., Vieytes M.R., Botana L.M. (2012). Comparative study of toxicological and cell cycle effects of okadaic acid and dinophysistoxin-2 in primary rat hepatocytes. Life Sci..

[24] Fernández D., Louzao M., Fraga M., Vilariño N., Vieytes M., Botana L. (2014). Experimental basis for the high oral toxicity of dinophysistoxin 1: A comparative study of DSP. Toxins.

[25] Del Campo M., Toledo H., Lagos N. (2013). Okadaic acid toxin at sublethal dose produced cell proliferation in gastric and colon epithelial cell lines. Mar. Drugs.

[26] Sérandour A.-L., Ledreux A., Morin B., Derick S., Augier E., Lanceleur R., Hamlaoui S., Moukha S., Furger C., Biré R. (2012). Collaborative study for the detection of toxic compounds in shellfish extracts using cell-based assays. Part I: Screening strategy and pre-validation study with lipophilic marine toxins. Anal. Bioanal. Chem..

[27] Leteurtre E., Gouyer V., Rousseau K., Moreau O., Barbat A., Swallow D., Huet G., Lesuffleur T. (2004). Differential mucin expression in colon carcinoma HT-29 clones with variable resistance to 5-fluorouracil and methotrexate. Biol. Cell.

[28] Wikman-Larhed A., Artursson P. (1995). Co-cultures of human intestinal goblet (HT29-H) and absorptive (Caco-2) cells for studies of drug and peptide absorption. Eur. J. Pharm. Sci..

[29] Haney S.A., LaPan P., Pan J., Zhang J. (2006). High-content screening moves to the front of the line. Drug Discov. Today.

[30] Thompson C.M., Fedorov Y., Brown D.D., Suh M., Proctor D.M., Kuriakose L., Haws L.C., Harris M.A. (2012). Assessment of Cr(VI)-induced cytotoxicity and genotoxicity using High Content Analysis. PLoS One.

[31] Scholzen T., Gerdes J. (2000). The Ki-67 protein: From the known and the unknown. J. Cell. Physiol..

[32] Porter A.G., Jänicke R.U. (1999). Emerging roles of caspase-3 in apoptosis. Cell Death Differ..

[33] Fu S., Yang Y., Tirtha D., Yen Y., Zhou B.-S., Zhou M.-M., Ohlmeyer M., Ko E.C., Cagan R., Rosenstein B.S. (2012). γ-H2AX kinetics as a novel approach to High Content Screening for small molecule radiosensitizers. PLoS One.

[34] Pasparakis M. (2012). Role of NF-κB in epithelial biology. Immunol. Rev..

[35] Rubiolo J.A., López-Alonso H., Vega F.V., Vieytes M.R., Botana L.M. (2011). Okadaic acid and dinophysis toxin 2 have differential toxicological effects in hepatic cell lines inducing cell cycle arrest, at G0/G1 or G2/M with aberrant mitosis depending on the cell line. Arch. Toxicol..

[36] Schmitz M.H.A., Held M., Janssens V., Hutchins J.R.A., Hudecz O., Ivanova E., Goris J., Trinkle-Mulcahy L., Lamond A.I., Poser I. (2010). Live-cell imaging RNAi screen identifies PP2A-B55alpha and importin-beta1 as key mitotic exit regulators in human cells. Nat. Cell Biol..

[37] Liu Y., Bodmer W.F. (2006). Analysis of P53 mutations and their expression in 56 colorectal cancer cell lines. Proc. Natl. Acad. Sci. USA.

[38] Green D.R., Kroemer G. (2009). Cytoplasmic functions of the tumour suppressor p53. Nature.

[39] Ehlers A., Scholz J., These A., Hessel S., Preiss-Weigert A., Lampen A. (2011). Analysis of the passage of the marine biotoxin okadaic acid through an* in vitro* human gut barrier. Toxicology.

[40] Kittler K., Preiss-Weigert A., These A. (2010). Identification strategy using combined mass spectrometric techniques for elucidation of phase I and phase II* in vitro* metabolites of lipophilic marine biotoxins. Anal. Chem..

[41] Guo F., An T., Rein K.S. (2010). The algal hepatoxoxin okadaic acid is a substrate for human cytochromes CYP3A4 and CYP3A5. Toxicon.

[42] Feng G., Ohmori Y., Chang P.-L. (2006). Production of chemokine CXCL1/KC by okadaic acid through the nuclear factor-κB pathway. Carcinogenesis.

[43] Karin M., Lin A. (2002). NF-kappaB at the crossroads of life and death. Nat. Immunol..

[44] Tinel A., Janssens S., Lippens S., Cuenin S., Logette E., Jaccard B., Quadroni M., Tschopp J. (2007). Autoproteolysis of PIDD marks the bifurcation between pro-death caspase-2 and pro-survival NF-kappaB pathway. EMBO J..

[45] Tubaro A., Sosa S., Altinier G., Soranzo M.R., Satake M., Della Loggia R., Yasumoto T. (2004). Short-term oral toxicity of homoyessotoxins, yessotoxin and okadaic acid in mice. Toxicon.

[46] Souid-Mensi G., Moukha S., Mobio T.A., Maaroufi K., Creppy E.E. (2008). The cytotoxicity and genotoxicity of okadaic acid are cell-line dependent. Toxicon Off. J. Int. Soc. Toxinol..

[47] Le Hégarat L., Fessard V., Poul J.M., Dragacci S., Sanders P. (2004). Marine toxin okadaic acid induces aneuploidy in CHO-K1 cells in presence of rat liver postmitochondrial fraction, revealed by cytokinesis-block micronucleus assay coupled to FISH. Environ. Toxicol..

[48] Traoré A., Baudrimont I., Ambaliou S., Dano S.D., Creppy E.E. (2001). DNA breaks and cell cycle arrest induced by okadaic acid in Caco-2 cells, a human colonic epithelial cell line. Arch. Toxicol..

[49] Lu C., Zhu F., Cho Y.-Y., Tang F., Zykova T., Ma W., Bode A.M., Dong Z. (2006). Cell apoptosis: Requirement of H2AX in DNA ladder formation, but not for the activation of caspase-3. Mol. Cell.

[50] Ravindran J., Gupta N., Agrawal M., Bala Bhaskar A.S., Lakshmana Rao P.V. (2011). Modulation of ROS/MAPK signaling pathways by okadaic acid leads to cell death via, mitochondrial mediated caspase-dependent mechanism. Apoptosis.

[51] Valdiglesias V., Laffon B., Pásaro E., Cemeli E., Anderson D., Méndez J. (2011). Induction of oxidative DNA damage by the marine toxin okadaic acid depends on human cell type. Toxicon Off. J. Int. Soc. Toxinol..

[52] Haneji T., Hirashima K., Teramachi J., Morimoto H. (2013). Okadaic acid activates the PKR pathway and induces apoptosis through PKR stimulation in MG63 osteoblast-like cells. Int. J. Oncol..

[53] Fujita M., Goto K., Yoshida K., Okamura H., Morimoto H., Kito S., Fukuda J., Haneji T. (2004). Okadaic acid stimulates expression of Fas receptor and Fas ligand by activation of nuclear factor kappa-B in human oral squamous carcinoma cells. Oral Oncol..

[54] Kitazumi I., Maseki Y., Nomura Y., Shimanuki A., Sugita Y., Tsukahara M. (2010). Okadaic acid induces DNA fragmentation via caspase-3-dependent and caspase-3-independent pathways in Chinese hamster ovary (CHO)-K1 cells. FEBS J..

[55] Millward T.A., Zolnierowicz S., Hemmings B.A. (1999). Regulation of protein kinase cascades by protein phosphatase 2A. Trends Biochem. Sci..

[56] Chang F., Steelman L.S., Shelton J.G., Lee J.T., Navolanic P.M., Blalock W.L., Franklin R., McCubrey J.A. (2003). Regulation of cell cycle progression and apoptosis by the Ras/Raf/MEK/ERK pathway (Review). Int. J. Oncol..

[57] Li H.-H., Cai X., Shouse G.P., Piluso L.G., Liu X. (2007). A specific PP2A regulatory subunit, B56γ, mediates DNA damage-induced dephosphorylation of p53 at Thr55. EMBO J..

[58] Zitvogel L., Kepp O., Galluzzi L., Kroemer G. (2012). Inflammasomes in carcinogenesis and anticancer immune responses. Nat. Immunol..

